# Cambodia achieved a high vaccination coverage for its population: A good example of a lower middle-income country

**DOI:** 10.7189/jogh.12.03088

**Published:** 2022-12-21

**Authors:** Ngoc Phuong Hong Tao, Dang Nguyen, Le Huu Nhat Minh, Veasna Duong, Chey Beaupha, Tareq AL Ahdal, Nguyen Tien Huy

**Affiliations:** 1 Chelsea High School, Chelsea, Michigan, USA; 2 Online Research Club, Nagasaki, Japan; 3 Department of Biomedical Engineering, University of South Florida, Tampa, Florida, USA; 4 International Ph.D. Program in Medicine, College of Medicine, Taipei Medical University, Taipei, Taiwan; 5 Virology Unit, Institut Pasteur du Cambodge, Institut Pasteur International Network, Phnom Penh, Cambodia; 6 National Authority for Combating Drugs (NACD), Phnom Penh, Cambodia; 7 Institute of Global Health (HIGH), Heidelberg University, Heidelberg, Germany; 8 School of Tropical Medicine and Global Health, Nagasaki University, Nagasaki, Japan

The COVID-19 global epidemic is a major catastrophe with disastrous negative health effects on the economy and society. Vaccination is a straightforward, effective, and risk-free option for guarding against COVID-19. Governments of many countries have implemented strategies in managing hazards and vaccination recommendations for their citizens. Gavi's COVID-19 Vaccines Global Access (COVAX) Advance Market Commitment (AMC) provided access to COVID-19 vaccinations to more than 90 countries, including low-income countries like Cambodia. The effective integration of vaccines in Cambodia has resulted in the saving of lives, the consolidation of the health care system, and a contribution to national progress toward economic recovery.

## PUBLIC HEALTH POLICIES IN CAMBODIA DURING COVID-19

**Figure 1:**
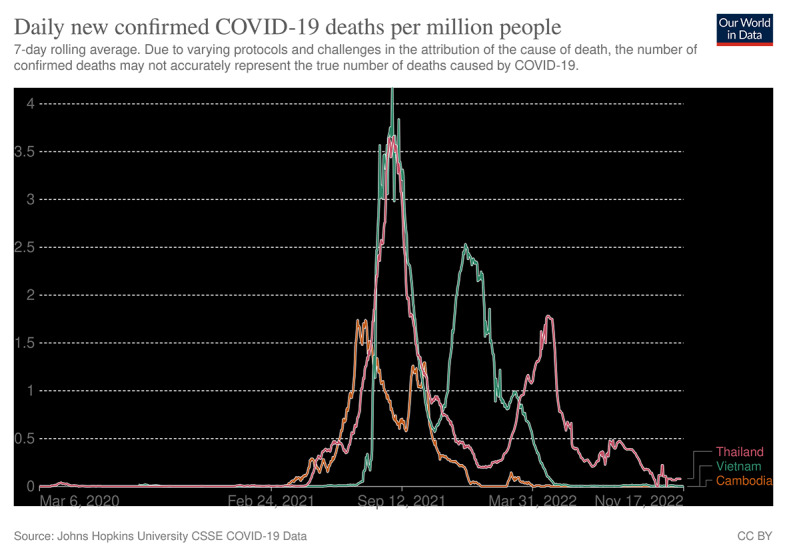
Daily new confirmed COVID-19 deaths per million people.

**Figure 2:**
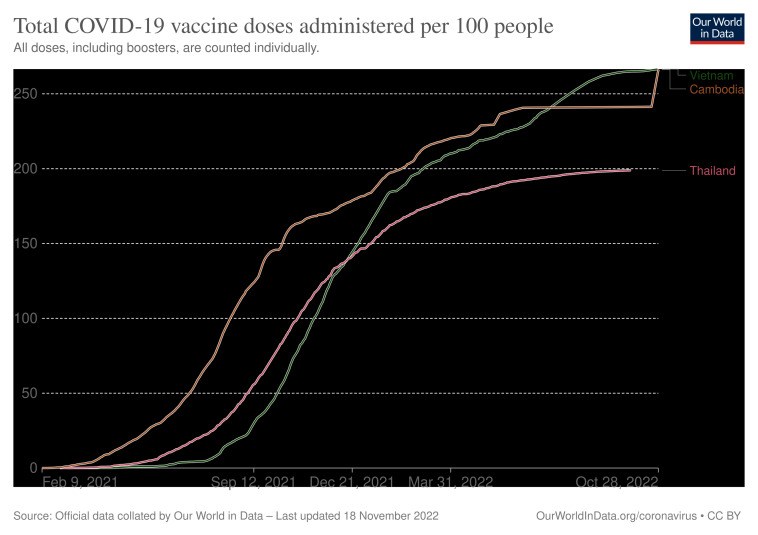
Total COVID-19 vaccine doses administered per 100 people.

The Cambodian Government has implemented multiple approaches to increase vaccine coverage. While many countries were hesitant to vaccines introducing early in the pandemic, Cambodia promptly purchased and approved the emergency use of Sinovac and Sinopharm vaccines from China and Covidshield from India since February 2021 [1], relatively earlier than neighbouring countries such as Thailand and Vietnam. Hence, from February 2021 to October 2022, the country acquired the highest number of COVID-19 vaccines administered and the lowest number of confirmed COVID-19 deaths compared to Thailand and Vietnam **(****Figure 1** and **Figure 2****)** [2,3]. Additionally, Cambodia received international donations of vaccines, medical equipment, and financial support, which demonstrated the commitment to providing sufficient doses to every citizen. The government prioritizes the development of medical facilities and human resources to support citizens in getting vaccinated. A national COVID-19 vaccination committee, more comprehensively organized with holistic approaches from the central government to local regions compared to neighbouring countries, was established to provide training for health care workers on the vaccination procedure and to timely distribute vaccines across the nation. Sampling laboratories, testing locations, and vaccination centres were constructed nationwide, with training plans focused on molecular diagnostics [4]. Volunteers including medical workers and medical students coordinated with the military personnel to enforce public health measures such as quarantines and lockdowns. The collaboration between local volunteers and officials helped promote vaccination which targeted marginalized indigenous minorities and migrants living near borders, who are often economically disadvantaged and have limited access to health services, by providing communication with the local community on the importance of receiving vaccination [5,6]. Although fewer people are receiving additional doses and boosters than primary doses post-COVID-19, the availability of extra mRNA vaccines may incentivize them to have third and fourth doses. These approaches could contribute to individual protection against the disease, prevention of COVID-19 transmission, and thereby alleviating the burden on the health care system.

These public health measures would have not succeeded if COVID-19 information had not been delivered efficiently. Messages of testing and promoting boosters were respectively integrated into mobile phone alerts when COVID-19 started and after the pandemic was relatively under control. To gain public trust and engage more individuals in the vaccination rollout, public authorities voluntarily got vaccinated [7].

## CAMPAIGNS TO INCREASE VACCINE COVERAGE AND ECONOMIC RECOVERY IN CAMBODIA POST-PANDEMIC

Along with its effective vaccination campaign during the peak of COVID-19, Cambodia continues to expand its vaccine coverage post-pandemic. The Cambodia Rapid Immunization Support Project was approved in February 2022, aiming to distribute vaccines inclusively to gender and socioeconomic background and to provide fully vaccinated individuals access to boosters and under-5-year-old children access to primary doses by 2023. Vaccination campaigns continue accelerating to at-risk and susceptible individuals, with house-to-house and after-hour services [8]. Additionally, Cambodia initiated the nationwide fifth dose campaign for individuals with at least 3 months of completion from their last dose. As of June 2022, Cambodia strengthens its public health measures to maintain disease control and mitigate negative effects on post-pandemic recovery. It emphasizes the utilization of surveillance systems with testing support provided by the World Health Organization (WHO), the United States Centers for Disease Control (US CDC), and the National Center for Health Promotion (NCHP). COVID-19 services, such as ventilators employment and site-specific training in oxygen therapy, were implemented in medical institutions through mentoring programs, and case definition has been inclusively expanded [8].

**Figure:**
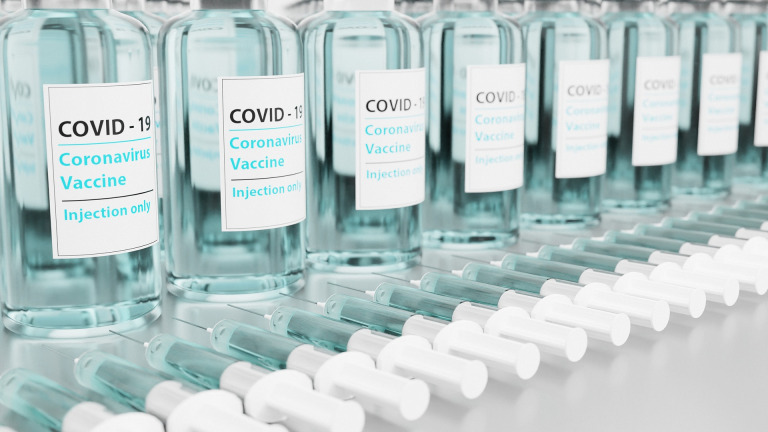
Photo: COVID-19 vaccines. Source: photo by Simon T (2022), available at: https://pixabay.com/photos/vaccine-vaccination-covid-19-5926664/ (free to use under the Pixabay license https://pixabay.com/service/license/).

Though Cambodia successfully executed vaccination programs and economic administration, it should address some critical issues to promote the long-term development of the health systems and economy. There has been a need to increase health care resources such as medical equipment, health institutions, and medical staff, and to update surveillance of COVID-19 positive cases, considering that Cambodia has a significantly low health capacity compared to the average of the Organization for Economic Cooperation and Development (OECD) countries. Additionally, it should foster diversity, shifting to greater value-added production in various business sectors, including agriculture, large-scale manufacturing, and tourism [9]. Looking toward the post-pandemic recovery, Cambodia partnered with the Asian Development Bank and the Greater Mekong Subregion to integrate strategies up to 2023, which will focus on enhancing the medical systems and confronting major challenges in the universality of health access and sustainable economic development.

## CONCLUSION

Approximately 25% of the global population had received 2 COVID-19 doses by the end of August 2021 [10]. As of August 2021, 88.35% of Cambodia’s 10 million prioritized adults have received the vaccination, and an intense program to deliver booster doses was initiated [11]. The rapid response of the Cambodian government, compared to other developing countries, is a successful attempt to alleviate the regional crisis caused by COVID-19. The outbreak has been well-managed, and the negative impacts resulting from the pandemic have been mitigated.
